# Points

**Published:** 1898-02

**Authors:** Wm. H. Steele


### COMMUNICATIONS.

### Points.

BY DR. WM. H. STEELE.

Read before the Northern Iowa Dental Society, September, 1897.

*Mr. President and Gentlemen:*

In casting about for a subject for this paper I decided to rake over the rubbish heap of "Has Beens," and resurrect one of our lost arts. Time was when every dentist had to be, to a great extent, his own instrument-maker, and before the advent of the dental engine when hand-instruments were used exclusively; all broken or worn-out instruments were carefully laid aside for a dull-day job, when they would be worked over into new points, tempered and polished. Of course some operators were more expert at this work than others, but almost every one who practiced dentistry could do the work, while to-day there is probably not one in five who knows how to work steel properly.

Instruments are now so cheap that it does not pay the busy practitioner to devote much of his valuable time to this work; but often a useful instrument is broken in an operation, or some special point is needed for a particular case, and it is very convenient to be able to do the work when necessary. Only the finest quality of steel should be used, and it must be manipulated in such manner as to retain its good qualities until the instrument is finished. The best quality of English tool steel or Stubbs steel drill rods, will prove satisfactory if properly worked.

*Forging.*—Great care should be taken not to overheat steel as it destroys the grain and makes it brittle. Small instruments should never be heated to a point that will raise scales while be-while being worked. In drawing a point all sides must be hammered alike, otherwise the point is liable to spring in tempering, as the side hammered most will have the finest grain. When a point has once been forged flat, do not attempt to hammer it back to a round or square, as such manipulation will spoil the instrument.

Steel should not be hammered after the color fades; until in the final heating, when it should be hammered to a dead black, and the last strokes should be on the cutting edge.

*Rough Dressing, Tempering.*—After the instrument is forged to shape, as well as can be with the hammer, it should be dressed up with suitable files, then tempered. For fine tempering I prefer night or a dark corner, in order to better watch the color. For drills heat the point to a cherry-red and dip in mercury; for excavators and small chisels, make a shallow tray $\frac{1}{8}$ in. deep and fill with the following: 1 oz. of wax and $\frac{1}{2}$ oz. rosin. Heat the point to a deep red and plunge into the composition. This method is preferable to tempering and drawing, for small instruments, as it is more certain and requires less skill to produce uniform results. For larger instruments, I prefer to temper and draw, using a bath made of sperm-oil and tallow; heat to a deep cherry red, dip the same as in a water bath and draw to a pale straw color. If a chisel is desired very hard on the cutting-edge, place that part of the instrument on a piece of cold steel, hold the shank in the flame and draw to a straw color, allowing the color to run no farther than to the portion resting on the steel.

*Finishing and Polishing.*—After the instruments are tempered they can be finished on the lathe with an Arkansas stone, and polished with crocus, using three grades, coarse, medium and fine; use the first two grades on cotton buff wheels, and the last one on a chamois buff.

*General Hints.*—Broaches, explorers and other small instruments can be annealed very soft and uniform by placing them in a piece of iron tubing; fill around with iron or brass filings, plug the ends of the tube, heat it gradually to a red heat, cover over with hot sand, and allow to cool over night. To temper small springs, heat to a cherry red and dip into water, then place in a dish and cover with oil, hold the dish over the flames until the oil ignites, then set away and let cool in the oil. In tempering in oil or water, always dip the instrument perpendicularly; if dipped on a slant, it is liable to cause the instrument to spring, especially if the bath is very cold. When tempering light work, the instrument must be handled quickly, for the edge where the best temper is required is the smallest part and cools first, often rendering the temper defective, just where it is desired most perfect. It is a good idea to have an assortment of blank instruments made up, dressed to a nice taper, annealed soft and laid away for an emergency; when needed the point can be tempered, and the remainder of the instrument being soft, it can readily be bent to reach and fit any cavity desired. By this means a large amount of hard work and valuable tooth substance can often be saved in excavating for filling.

